# Translation, Cultural Adaptation, and Reliability of the 15D Portuguese Version: A Generic Health-Related Quality of Life (HRQoL) Instrument

**DOI:** 10.3390/healthcare11081099

**Published:** 2023-04-12

**Authors:** Soraia Ferreira, Harri Sintonen, Armando Raimundo, Nuno Batalha, María Mendoza-Muñoz, Jorge Perez-Gomez, Jose A. Parraca

**Affiliations:** 1 Departamento de Desporto e Saúde, Escola de Saúde e Desenvolvimento Humano, Universidade de Évora, 7004-516 Évora, Portugal; 2 Comprehensive Health Research Centre (CHRC), Universidade de Évora, Largo dos Colegiais, 7004-516 Évora, Portugal; 3 Department of Public Health, University of Helsinki, 00100 Helsinki, Finland; 4 Research Group on Physical and Health Literacy and Health-Related Quality of Life (PHYQOL), Faculty of Sport Sciences, University of Extremadura, 10003 Caceres, Spain; 5 Health, Economy, Motricity, and Education (HEME) Research Group, Faculty of Sport Sciences, The University of Extremadura, 10003 Cáceres, Spain

**Keywords:** quality of life, health survey, Portugal, population

## Abstract

(1) Background: Purpose: The assessment of quality of life is essential to the human condition and can be measured through questionnaires. This study aims to translate and culturally adapt the 15D questionnaire to assess the population’s quality of life, as well as explore its relative reliability and internal consistency; (2) Methods: The translation and cultural adaptation of the 15D questionnaire was carried out independently, considering two translations. The synthesis version was applied to eight subjects, distributed by gender. Cognitive interviews were conducted to observe clarity, acceptability, and familiarity with the version of the questionnaire. The final version of the questionnaire, in Portuguese, was again translated into the official language by two translators who had never had contact with the questionnaire. To assess the test-retest reliability and internal consistency of the 15D questionnaire, 43 participants were interviewed; (3) Results: Participants indicated that they had some doubts about dimensions, breathing, and discomfort and symptoms; however, as there were no suggestions for change, the questionnaire had no changes. Items were clear and understandable. Internal consistency was observed using Cronbach’s alpha, with values between 0.76 and 0.98. The test-retest reliability values were between 0.77 and 0.97; and (4) Conclusions: The Portuguese version of the 15D questionnaire was proved to be equivalent to the English version and to be reliable for the Portuguese population. This instrument is easy to access and apply.

## 1. Introduction

Quality of life (QoL) may be affected by social relationships, the environment, physical and mental health, and personal beliefs. The World Health Organization (WHO) defines QoL as the individual’s perception of his/her position in life, which is present in different contexts, according to individual goals, concerns, and expectations [1]. Over the past few years, several questionnaires have been developed to measure QoL in different dimensions. Some of these questionnaires have a Portuguese translated version, such as the World Health Organization Quality of Life Instruments—Bref (WHOQOL-BREF) [2], the Euroqol 5D (EQ-5D) [3], the Quality of Life Scale (QOLS), and the Short Form Health Survey (SF-36) [4]. These questionnaires address several domains of QoL and are reliable for different scores. In this sense, a subcomponent of global QoL related to individuals’ health status is known as health-related quality of life (HRQoL), which focuses on the patient’s own perception of health and ability to function as a result of the health status or illness experience [5]. Despite the existence of several questionnaires translated into Portuguese, the need arose to find a questionnaire that was quick to apply and that addressed several domains of HRQoL. A questionnaire widely used in research has been the 15D [6,7], which aims to assess the HRQoL of a population and is a very comprehensive questionnaire. This questionnaire in English was developed in Finland and is composed of 15 dimensions/items which are based on mobility, vision, hearing, breathing, sleeping, eating, speech, excretion, usual activities, mental function, discomfort and symptoms, depression, anxiety, vitality, and sexual activity [7]. It is a self-administered questionnaire, and, in each dimension, only one of the five levels of functional status can be selected. According to the answers, participants are classified as to their health condition, with 1 representing the best possible health condition and 5 the worst possible health condition. Previous studies have shown that this questionnaire is easy to apply and valid for tracking the quality of life in different types of populations [8,9]. However, although the 15D questionnaire is available in several languages, such as Arabic, Czech, Danish, English, German, Greek, Hebrew, Japanese, Norwegian, Russian, Serbian, Spanish, and Swedish [10], it is not yet available in Portuguese of Portugal.

Thus, the present study aims to perform and assess the translation and linguistic and cultural adaptation of the 15D questionnaire into the Portuguese language of Portugal, as well as exploring its relative reliability and internal consistency.

## 2. Materials and Methods

### 2.1. Ethics Approval

The Ethics Committee of University of Evora approved this study (protocol code: GD/10815/2017). This study was performed in line with the principles of the Declaration of Helsinki.

### 2.2. Intrument

15D. It is a self-administered instrument that aims to assess the HRQoL of a population. The questionnaire is composed of 15 dimensions/items (mobility, vision, hearing, breathing, sleep, eating, speech, excretion, usual activities, mental function, discomfort and symptoms, depression, anxiety, vitality, and sexual activity) [7], which have five levels of functional status, from which only one can be selected, where 1 represents the best possible health status and 5 the worst possible health status.

This instrument has been translated into multiple languages and has proven internal consistency in populations such as Greek (alfa de Cronbach = 0.835) or Turkish (Cronbach’s alpha= 0.89) [8,10]. Moreover, previous studies have shown that this questionnaire is easy to administer and valid for monitoring quality of life in different types of populations, such as coronary artery disease or Parkinson’s disease [8,9].

### 2.3. Translation and Cultural Adaptation

The methodology applied was similar to that of previous studies [11,12], focusing on direct and reverse translation and the use of cognitive interviews. All participants were informed of the objectives of the study and gave informed consent for participation. The study was approved by the Ethics Committee for Research in Health and Human Welfare Areas of the University of Évora.

Phase 1—Translation of the 15D questionnaire into Portuguese.

Initially, we performed two translations of the English version of the 15D into Portuguese of Portugal. This translation was carried out by two independent translators (T1 and T2) from different professional areas (languages and sports science), with fluent English and native speakers of the target language. The translators discussed the two versions, arriving at a consensus version (version 1), where the aim was to obtain a translation version that was similar in the response options, the application instructions and the concepts and semantics of the dimensions.

Phase 2—Cognitive interventions to test the questionnaire in patients.

To assess the clarity of the instrument and determine whether the translated version of the questionnaire was easy to understand, it was applied to adults. After the application of the questionnaire, a cognitive interview was conducted with the participants, where feedback was requested on possible misunderstandings/errors and suggestions for improvement, to improve the questionnaire. Eight adults participated in the interviews and all were applied identically by the same interviewer, a native Portuguese speaker. The data obtained were grouped according to gender, age, and disease.

The interviews were based on 4 aspects: (a) understanding the instructions and answer options; (b) difficulty in understanding the different items of the questionnaire, which were assessed dichotomously, where 1 represented that the item was clear and understandable and 2 that there was difficulty in understanding it; (c) assessment, through a numerical scale, of whether the items of the questionnaire were easy to understand. The numerical scale was from 0 to 10, where 0 meant that the item was very easy to understand and 10 meant the item was difficult to understand; and (d) analysis of the interpretations made of the different items, by the interviewees. The participants were asked to suggest improvements in the questionnaire and to indicate, in their own words, what they understood of each item, and to rewrite it.

Modifications were proposed whenever a problem of understanding arose. At the end of this process, Portuguese version 2 was translated into Portuguese.

Characterization of the Cognitive Interviews Sample.

Table 1 shows the characteristics of the participants who took part in the cognitive interviews. This sample consisted of 8 adults, 4 males and 4 females, with an average age of 69.87 years. As for schooling, most of the participants have completed basic education, two of the interviewees have higher education grades and two others have secondary education grade (Table 1).

Phase 3—Translation from Portuguese to English.

Version 2 of the 15D was translated into English with the help of two translators fluent in English and with no health background. They had never seen the original English version of the questionnaire. The version that was proposed by the translators was reviewed by a group of experts composed of the project leader, the surveyor, and the translators. This review aimed to understand if there were any misunderstandings or inconsistencies in the translation process of the questionnaire. The author’s approval of the use of the questionnaire was another aspect to be taken into consideration in this last revision, and this last version was approved for use.

### 2.4. Psychometric Properties

The test-retest reliability and internal consistency of the Portuguese version of the 15D questionnaire were analyzed taking into account 43 participants (Table 2). This is a convenience sample, referred by family doctors from the Lusitânia Family Health Unit in Évora. The questionnaire was applied twice, at two different times, with an interval of four to seven days. The questionnaire was applied through interviews with the participants, which took place in person.

Table 2 shows the characteristics of the participants who took part in the test-retest process, who had a mean age of 63.81 and 62.79% were male.

### 2.5. Statistical Analysis

All variables were tested for normality with the Shapiro-Wilks test. Internal consistency (IC) was studied using Cronbach’s alpha, with values below 0.6 being unacceptable, values between 0.6 and 0.7 presenting a weak IC, values between 0.7 and 0.8 a reasonable IC, values between 0.8 and 0.9 a good IC, and values above 0.9 a very good IC [13]. Relative test-retest reliability was studied using the intraclass correlation coefficient (ICC) and the cut-off values used were: ICC < 0.5 poor reliability; 0.5 to 0.74 moderate reliability; 0.75 to 0.90 good reliability; and >0.90 excellent reliability [14]. Absolute reliability was studied using the standard error of measurement (SEM) and the minimum detectable change (MDC). The EPM is calculated by the formula EPM = SD√(1 − ICC), with smaller values indicating higher reliability [15]. The MMD was calculated for a 95% confidence level by the formula MMD95 = 1.96 × √2 × SEM [16]. We analyzed the data using SPSS (Statistical Package for Social Sciences) software, version 25.0.

## 3. Results

### 3.1. Translation and Cultural Adaptation

Phase 1—translation of the 15D questionnaire from English into Portuguese.

Phase one of the study aimed to obtain the first version of the 15D questionnaire in its Portuguese version after translation by two independent translators. After a discussion regarding these two preliminary versions, some words, concepts and terms in the first version of the questionnaire were changed.

Throughout the various dimensions, the terms “able” and “can” were defined with consistency. “Able” was defined as “ser capaz” and “can” as “consigo”.

In the first dimension, the term “subir e descer” was added before the word “escadas”. The term “recurso a equipamento de apoio” was used instead of “appliance”. In dimension 7, the term “emito murmúrios” was replaced by “murmuro”. In dimension 13, the word “Angustia” was replaced by “Ansiedade”, and in the mental function dimension, the terms “pensar de forma clara” instead of “claramente” and “lógica” instead of “logicamente” were used.

In the hearing dimension, the term “hearing aid” was changed to “aparelho auditivo”, because in Portugal it is a more commonly used term.

In the fourth dimension, concerning breathing, the term “heavy work” was translated as “intenso” and “sports” as “prática desportiva”, because sport includes a competitive component.

In the eating dimension, it was necessary to find a synonym for the words “slowly”, “clumsity” and “shakily”, and the words “devagar”, “desajeitado” and “trémulo”, were used, respectively.

In dimension 8, the title used was excretion, because it was more directed to the context of the questions, and the word “micção” was replaced by “urinar”

In the dimension usual activities, the word “emprego” was replaced by “trabalho”, because it is a broader term.

In the Vitality dimension, the word “exausto” has been replaced by “abatido”, because the word exhausted means as tired as possible. The word was only kept in the last option.

Phase 2—Use of cognitive interventions to test the questionnaire on patients.

**Figure 1:**
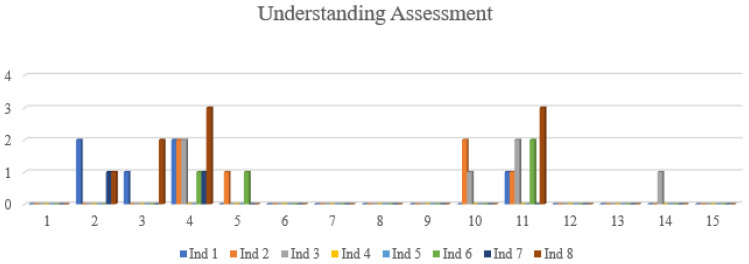
Evaluation of questionnaire comprehension in cognitive interviews.

Figure 1 shows the response of the eight participants for the 15 dimensions in relation to their understanding. Thus, in the evaluation of the respondents’ answers for version 1, it was observed that there was a variation between 0 and 3 regarding the understanding of the 15 dimensions, where 0 meant that the item was very easy to understand and, as it increased to 10, that the item is difficult to understand. Respondents indicated 7 dimensions as being slightly difficult to understand; however, the dimensions breathing and discomfort and symptoms have the highest values. The dimensions of mobility, eating, speech, excretion, ritual activities, depression, anxiety, and sexual activity presented an excellent compression by the interviewees. Although some dimensions raised doubts, no changes were made to the questionnaire, because the participants did not make any suggestions.

Phase 3—Translation from Portuguese of Portugal into English.

The translation from Portuguese of Portugal into English was carried out with the help of a professional English translator, fluent in Portuguese of Portugal. This translator translated the version into English, without ever having access to the English version of the 15D. After translation, the document was reviewed by translators, the original author, physicians, and the researcher who would apply the questionnaire. As no inconsistency was found in the translation of the questionnaire, and as the application instructions, the semantics of the dimensions, and concepts were similar, the original author approved the use of this translation.

Phase 4—Transformation of the 15D score into Utility 5.

According to the methodology mentioned by Sintonen (1995), the 15D score was transformed into utility.

- Mobility (1 = 1) (2 = 0.7129) (3 = 0.4729) (4 = 0.2526) (5 = 0.0780).
- Vision (1 = 1) (2 = 0.7840) (3 = 0.4901) (4 = 0.3137) (5 = 0.1089).
- Hearing (1 = 1) (2 = 0.7497) (3 = 0.4611) (4 = 0.2353) (5 = 0.1003).
- Breathing (1 = 1) (2 = 0.6976) (3 = 0.4771) (4 = 0.2581) (5 = 0.0879).
- Sleeping (1 = 1) (2 = 0.7615) (3 = 0.5124) (4 = 0.3015) (5 = 0.1115).
- Eating (1 = 1) (2 = 0.6462) (3 = 0.4267) (4 = 0.1984) (5 = 0.0710).
- Speech (1 = 1) (2 = 0.7033) (3 = 0.4322) (4 = 0.2471) (5 = 0.1298).
- Elimination (1 = 1) (2 = 0.6845) (3 = 0.3958) (4 = 0.1764) (5 = 0.0558).
- Usual Activities (1 = 1) (2 = 0.7210) (3 = 0.4133) (4 = 0.2182) (5 = 0.0785).
- Mental Function (1 = 1) (2 = 0.6434) (3 = 0.3750) (4 = 0.1956) (5 = 0.0489).
- Discomfort and Symptoms (1 = 1) (2 = 0.7024) (3 = 0.3960) (4 = 0.2083) (5 = 0.0617).
- Depression (1 = 1) (2 = 0.7651) (3 = 0.5148) (4 = 0.3053) (5 = 0.1576).
- Distress (1 = 1) (2 = 0.7251) (3 = 0.4786) (4 = 0.2633) (5 = 0.1255).
- Vitality (1 = 1) (2 = 0.7713) (3 = 0.5152) (4 = 0.2957) (5 = 0.1253).
- Sexual Activity (1 = 1) (2 = 0.7095) (3 = 0.4424) (4 = 0.2486) (5 = 0.1318).

Descriptive statistics and internal consistency:

In both moments of the 15D questionnaire (test and retest), more than 80% of the participants selected level 1 or 2, except in the dimensions of discomfort and symptoms, anxiety, and sexual activity, as shown in the Table 3. In the dimension of hearing, more than 80% of the participants selected level 1 and in the dimension of eating, the same level was selected by more than 90% of the participants. Cronbach’s α was only below 0.800 in the dimensions of discomfort and symptoms and eating.

### 3.2. Psychometric Properties

Table 4 shows the data obtained in the test-retests, as well as the reliability and internal consistency. The data belong to 43 adults (27 men and 16 women) with a mean age of 63.81 ± 9.5 (mean ± SD). Concerning relative reliability, the ICC shows excellent reliability for the dimensions of hearing and sexual activity, with a value higher than 0.9. The remaining dimensions show good reliability, with values between 0.77 and 0.89. The internal consistency values are between 0.76 and 0.98 for the 15 dimensions, meaning that there is good consistency (Table 4). The absolute reliability EPM values are acceptable, with the highest value corresponding to 24.6%. In some dimensions, the MDR values are very high, meaning that many improvements are necessary for clinically relevant changes to occur.

## 4. Discussion

In the present research, we assessed the translation and linguistic adaptation of the 15D into the Portuguese language of Portugal. The translation process implies a cross-cultural adaptation, to use expressions from the country; however, an accurate translation of the health measures is necessary [7,17]. The methodology used in the translation process respected the concepts, terms, and semantics of the English version of the 15D. Previous studies have translated the 15D questionnaire into other languages, showing a high ICC and internal consistency [18,19].

Concerning the percentage of participants who selected each dimension, we highlight the fact that approximately 85% of the participants selected levels 1, 2 or 3 for 14 dimensions. The dimension of sexual activity presents the highest percentage of participants who selected level 5, this being higher than 10%. These results show that the most affected dimension of QoL is sexual activity. Our study data are in line with a study carried out in Japan, where the results of the sexual activity dimension are the worst, with levels 4 and 5 standing out for more than 10% of the participants. As for the remaining 14 dimensions, approximately 90% of the participants selected level 1 or 2 [12]. On the other hand, a study developed with the Greek population obtained lower results than the present study, with between 10% and 60% of the participants selecting levels 3, 4 and 5 of each dimension [8].

In the present investigation, Cronbach’s α values concerning the dimensions of the questionnaire ranged between 0.763 and 0.976. Only the items discomfort and symptoms and eating had an IC with values lower than 0.8, which was reasonable. All other variables had a good or very good IC. However, the IC of the 15D questionnaire of the present investigation is confirmed as the values obtained are higher than 0.7. On the other hand, our results were slightly lower than those of previous studies which translated the 15D questionnaire into Greek and Turkish, where the IC values were higher than 0.81 and 0.82, respectively [8,10].

In the present study, the ICC was higher than 0.76, showing good reliability in all dimensions. The only dimensions which presented excellent reliability were hearing and sexual activity. Contrary to the values obtained, Okamoto’s study presented lower ICC values, varying between 0.44 and 0.72. Despite the difference in values between both studies, these can be justified by the advanced age of the participants, as the ages ranged between 65 and 89 years old [12]. On the other hand, a study carried out with the Turkish population presented ICC values higher than 0.8 [10].

Absolute reliability is observed through EPM and MDR. The EPM values present high reliability, all values being very close to 0. The dimensions of sleeping, usual activities, mental function, discomfort and symptoms, anxiety, and sexual activity present the highest EPM values, being the respective values of 0.112, 0.099, 0.098, 0.119, 0.96, and 0.168. In these dimensions, the percentage of participants presenting these values is higher than 10%. The highest percentage is observed in the anxiety dimension, where 22.13% of the total population presents higher values. According to the measurement accuracy requirements, the EPM of anxiety could be considered unacceptable, because the value was higher than the SD / 2 criterion. In the %MDR values, the dimensions of anxiety and sexual activity presented values higher than 60%, which means that for participants to have clinically relevant modifications they should improve their score by more than 60%, compared to the present results. Thus, for a clinically relevant modification in the questionnaire score to occur, participants should improve the anxiety value by 0.265 and the sexual activity value by 0.465.

This study presented the translation and cultural adaptation of the 15D for the first time into Portuguese of Portugal, and assessed its relative reliability and internal consistency, but did not assess content, concurrent, or construct validity, so future studies could study these parameters (reliability and validity) of the 15D questionnaire for different types of populations, such as different sexes, age groups, different pathologies or socio-economic groups. In addition, factor analysis was not tested. Therefore, other studies using this version could consider factor analysis in their research.

Furthermore, the present research presented a limitation regarding the sample because only 43 participants performed the test-retest. The absence of different age groups could be considered another limitation.

## 5. Conclusions

Based on the lack of a reliable adaptation of the 15D questionnaire into Portuguese, a translation, cultural adaptation, and reliability process was carried out. Thus, results show the 15D questionnaire in the Portuguese version is reliable to evaluate the HRQoL in the Portuguese population. This instrument could help collect data on the population’s quality of life, being a tool of easy access, no cost, and quick application.

## Figures and Tables

**Table 1:** Characterization of the Cognitive Interviews sample.

Demographic Variables	Mean (SD)	N (N = 8)	%
Age (years)	69.87 (14.55)	-	-
Education Grade Basic	-	4	50.0
Secondary	-	2	25.0
Higher	-	2	25.0
Gender	-		
Male		4	50.0
Female	-	4	50.0
Weight (kg)	82.81 (13.38)	-	-
Height (m)	1.67 (0.10)	-	-
BMI (kg/m^2^)	29.33 (2.78)	-	-
With Pathologies	-	4	50.0
Healthy	-	4	50.0

SD: standard deviation; BMI: body mass index.

**Table 2:** Demographic variables of the sample that participated in the internal consistency and test-retest reliability study.

Demographic Variables	Mean (SD)	N (N = 43)	%
Age (years)	63.81 (9.5)	-	-
Gender			
Male	-	27	62.79
Female	-	16	37.21
Weight (kg)	80.23 (18.70)	-	-
Height (m)	1.64 (0.11)	-	-
BMI (kg/m^2^)	29.47 (4.55)	-	-

SD: standard deviation; BMI: body mass index.

**Table 3:** Statistical description and internal consistency of the 15D.

	Response Frequency (%)										Cronbach’s α
	Test					Re-Test					
	1	2	3	4	5	1	2	3	4	5	
Mobility	69.8	16.3	14.0	0.0	0.0	67.4	18.6	14.0	0.0	0.0	0.860
Vision	62.8	20.9	16.3	0.0	0.0	69.8	16.3	14.0	0.0	0.0	0.886
Hearing	81.4	4.7	14.0	0.0	0.0	81.4	7.0	7.0	4.7	0.0	0.929
Breathing	62.8	30.2	2.3	2.3	2.3	58.1	32.6	0.0	9.3	0.0	0.897
Sleeping	51.2	30.2	7.0	7.0	4.7	58.1	25.6	9.3	2.3	4.7	0.800
Eating	95.3	4.7	0.0	0.0	0.0	97.7	2.3	0.0	0.0	0.0	0.796
Speech	86.0	14.0	0.0	0.0	0.0	79.1	18.6	2.3	0.0	0.0	0.864
Elimination	69.8	25.6	4.7	0.0	0.0	69.8	25.6	4.7	0.0	0.0	0.872
Usual Activities	58.1	23.3	11.6	7.0	0.0	65.1	20.9	7.0	2.3	0.0	0.855
Mental Function	53.5	37.2	9.3	0.0	0.0	53.5	32.6	11.6	2.3	0.0	0.821
Discomfort and Symptoms	39.5	41.9	14.0	4.7	0.0	48.8	30.2	18.6	2.3	0.0	0.763
Depression	69.8	23.3	4.7	2.3	0.0	67.4	27.9	2.3	2.3	0.0	0.883
Distress	44.2	37.2	9.3	9.3	0.0	46.5	32.6	14.0	7.0	0.0	0.837
Vitality	65.1	23.3	9.3	2.3	0.0	60.5	27.9	9.3	2.3	0.0	0.834
Sexual Activity	41.9	18.6	16.3	9.3	14.0	46.5	18.6	16.3	7.0	11.6	0.976

**Table 4:** Mean and Standard Deviation, Reliability and Internal Consistency.

	Test Mean (SD)	Re-Test Mean (SD)	*p*	ICC (95% CI)	SEM	%SEM	SDR	%SDR
Mobility	0.879 (0.196)	0.873 (0.197)	0.783	0.863 (0.748 to 0.925)	0.073	8.303	0.202	23.014
Vision	0.871 (0.191)	0.893 (0.182)	0.301	0.885 (0.789 to 0.938)	0.063	7.171	0.175	19.876
Hearing	0.913 (0.191)	0.909 (0.211)	0.713	0.930 (0.872 to 0.962)	0.053	5.837	0.147	16.181
Breathing	0.858 (0.216)	0.832 (0.232)	0.199	0.896 (0.810 to 0.944)	0.072	8.549	0.200	23.696
Sleeping	0.803 (0.260)	0.836 (0.243)	0.452	0.800 (0.632 to 0.891)	0.112	13.725	0.312	38.043
Eating	0.983 (0.075)	0.991 (0.53)	0.317	0.796 (0.625 to 0.889)	0.050	5.039	0.138	13.969
Speech	0.958 (0.104)	0.931 (0.140)	0.059	0.854 (0.731 to 0.921)	0.047	4.936	0.129	13.681
Elimination	0.891 (0.177)	0.891 (0.177)	1.000	0.875 (0.770 to 0.932)	0.063	7.023	0.173	19.468
Usual Activities	0.812 (0.259)	0.845 (0.257)	0.279	0.854 (0.731 to 0.922)	0.099	11.899	0.273	32.982
Mental Function	0.809 (0.219)	0.787 (0.246)	0.673	0.824 (0.674 to 0.905)	0.098	12.223	0.270	33.880
Discomfort and Symptoms	0.754 (0.241)	0.781 (0.251)	0.834	0.766 (0.566 to 0.874)	0.119	15.505	0.330	42.977
Depression	0.906 (0.164)	0.907 (0.154)	0.763	0.885 (0.790 to 0.938)	0.054	5.948	0.149	16.487
Distress	0.780 (0.239)	0.786 (0.236)	1.000	0.840 (0.707 to 0.913)	0.096	22.130	0.265	61.340
Vitality	0.885 (0.182)	0.874 (0.181)	0.783	0.837 (0.700 to 0.911)	0.073	8.332	0.203	23.094
Sexual Activity	0.664 (0.337)	0.701 (0.326)	0.064	0.973 (0.950 to 0.985)	0.168	24.575	0.465	68.120

ICC: Intraclass Correlation Coefficient; CI: Confidence Interval; SEM: Standard Error Measurement; SRD: Small Real Difference.

## Data Availability

The datasets are available through the corresponding author upon reasonable request.
